# Facile Strategy on Hydrophilic Modification of Poly(ε-caprolactone) Scaffolds for Assisting Tissue-Engineered Meniscus Constructs *In Vitro*

**DOI:** 10.3389/fphar.2020.00471

**Published:** 2020-05-01

**Authors:** Zhu-Xing Zhou, You-Rong Chen, Ji-Ying Zhang, Dong Jiang, Fu-Zhen Yuan, Zi-Mu Mao, Fei Yang, Wen-Bo Jiang, Xing Wang, Jia-Kuo Yu

**Affiliations:** ^1^Knee Surgery Department of the Institute of Sports Medicine, Peking University Third Hospital, Beijing, China; ^2^Beijing National Laboratory for Molecular Sciences, State Key Laboratory of Polymer Physics & Chemistry, Institute of Chemistry, Chinese Academy of Sciences, Beijing, China; ^3^University of Chinese Academy of Sciences, Beijing, China; ^4^Clinical Translational R&D Center of 3D Printing Technology, Shanghai Ninth People's Hospital, Shanghai Jiao Tong University School of Medicine, Shanghai, China

**Keywords:** tissue-engineered meniscus, poly(ε-caprolactone), fused deposition modeling, three-dimension printing, scaffold, hydrophilic modification, regenerative medicine

## Abstract

Poly(ε-caprolactone) (PCL) derived scaffolds have been extensively explored in the field of tissue-engineered meniscus (TEM) originating from their good biosafety and biomechanical properties. However, the poor intrinsic hydrophobicity severely hindered their wide applications for the scaffold-assisted tissue regeneration. Herein, we developed a simple strategy on surface modification of three-dimensional (3D) PCL scaffolds *via* a simply soaking treatment of sodium hydroxide (NaOH) solutions to increase the hydrophilicity and roughness of scaffolds' surfaces. We investigated the effect of hydrolysis degree mediated by NaOH solutions on mechanical properties of 3D scaffolds, considering the importance of scaffolds' resistance to internal force. We also investigated and analyzed the biological performances of mesenchymal stromal cells (MSCs) and meniscal fibrocartilage cells (MFCs) onto the scaffolds treated or untreated by NaOH solutions. The results indicated that hydrophilic modification could improve the proliferation and attachment of cells on the scaffolds. After careful screening process condition, structural fabrication, and performance optimization, these modified PCL scaffolds possessed roughened surfaces with inherent hierarchical pores, enhanced hydrophilicity and preferable biological performances, thus exhibiting the favorable advantages on the proliferation and adhesion of seeded cells for TEM. Therefore, this feasible hydrophilic modification method is not only beneficial to promote smarter biomedical scaffold materials but also show great application prospect in tissue engineering meniscus with tunable architectures and desired functionalities.

## Introduction

Traumatic meniscal disorder is one of the most common types of orthopedic injuries in many people of all ages (Beals et al., 2016). It was estimated that the number of patients in need of meniscal surgery was over 1.5 million until 2014 in Europe and US (Moran et al., 2015). Traditionally, arthroscopic surgery in clinical practice was performed to relieve symptoms and improve outcomes, especially, meniscal lesions in avascular region are irreversible and difficult to be restored, which often requires partial and total meniscectomy (Rongen et al., 2014; Roos and Thorlund, 2017; Thorlund et al., 2017). Up to date, physicians and researchers have been aware of the important role of menisci in knee joints, including the protective effect on cartilage, absorbing the shock, and maintaining the stability of the knees (Badlani et al., 2013). Therefore, a number of measures are devised to save meniscal tissues or replace the irreparable meniscal issues rather than simple removal of them.

One of the intellectual strategies is meniscal regeneration enhanced by tissue-engineered technique, in which meniscal scaffolds fabricated by biocompatible materials are loaded with living seeded cells (Petri et al., 2012; Zhang et al., 2017). Materials for scaffold's fabrication in that strategy play a vital role in scaffold-guided tissue regeneration, particularly for tissue-engineered meniscus (TEM), which are not only supposed to resist the load from femur and tibia with strong mechanical properties, but also provide a suitable microenvironment for seeded cells to reside. Based on those requirements, the emerging biodegradable macromolecular materials with tunable biophysical and biochemical properties promote the development of scaffolds for TEM dramatically. In the past few decades, a number of recent studies evaluated and published the results of biocompatibilities, biomechanical properties, and replacing functions of synthetic materials for TEM (Hannink et al., 2011; Verdonk et al., 2011; Esposito et al., 2013; Lee et al., 2014; Kwak et al., 2017; Moradi et al., 2017). In those materials, poly(ε-caprolactone) (PCL), as one of the aliphatic polyesters, possesses thermal plasticity and shapeability that can be molded into different three-dimensional (3D) scaffolds with designed geometric shape, degrading into nontoxic products after implantation into host (Oh et al., 2007; Zhang et al., 2017). More importantly, it had been an FDA-approved implantable biomaterial (Neufurth et al., 2017), which suggested its fitness for the tissue-engineered applications. In our recent research, we found that 3D-printed PCL scaffolds combined with biophysical and biochemical stimuli could contribute to well-regenerated and anisotropic menisci with better mechanical properties and protective effects on cartilage (Zhang et al., 2019). Thus, PCL-based TEM attracts wide attention and exhibits a promising prospect to realize meniscal regeneration and therapeutic potential of TEM applications.

However, similar to other aliphatic polymers with the intrinsic hydrophobicity, the hydrophilicity of the PCL should be improved although it has many abovementioned advantages and bright application prospects. When it comes to hydrophobicity, it leads to the lack of recognition site attached by cells on the surface of scaffold, impeding the cellular behavior on scaffold (Neufurth et al., 2017). Some strategies of hydrophilic modification such as protein coating, cold plasma treatment, and chemical etching are conducted to achieve well-attached property for scaffold (Ma et al., 2007). Given the time-consuming, complexity, and expensiveness, some of abovementioned techniques are not practical. Therefore, a simple method on the hydrophilic development of PCL scaffolds is urgent and imperative in the field of TEM.

**Figure 1:**
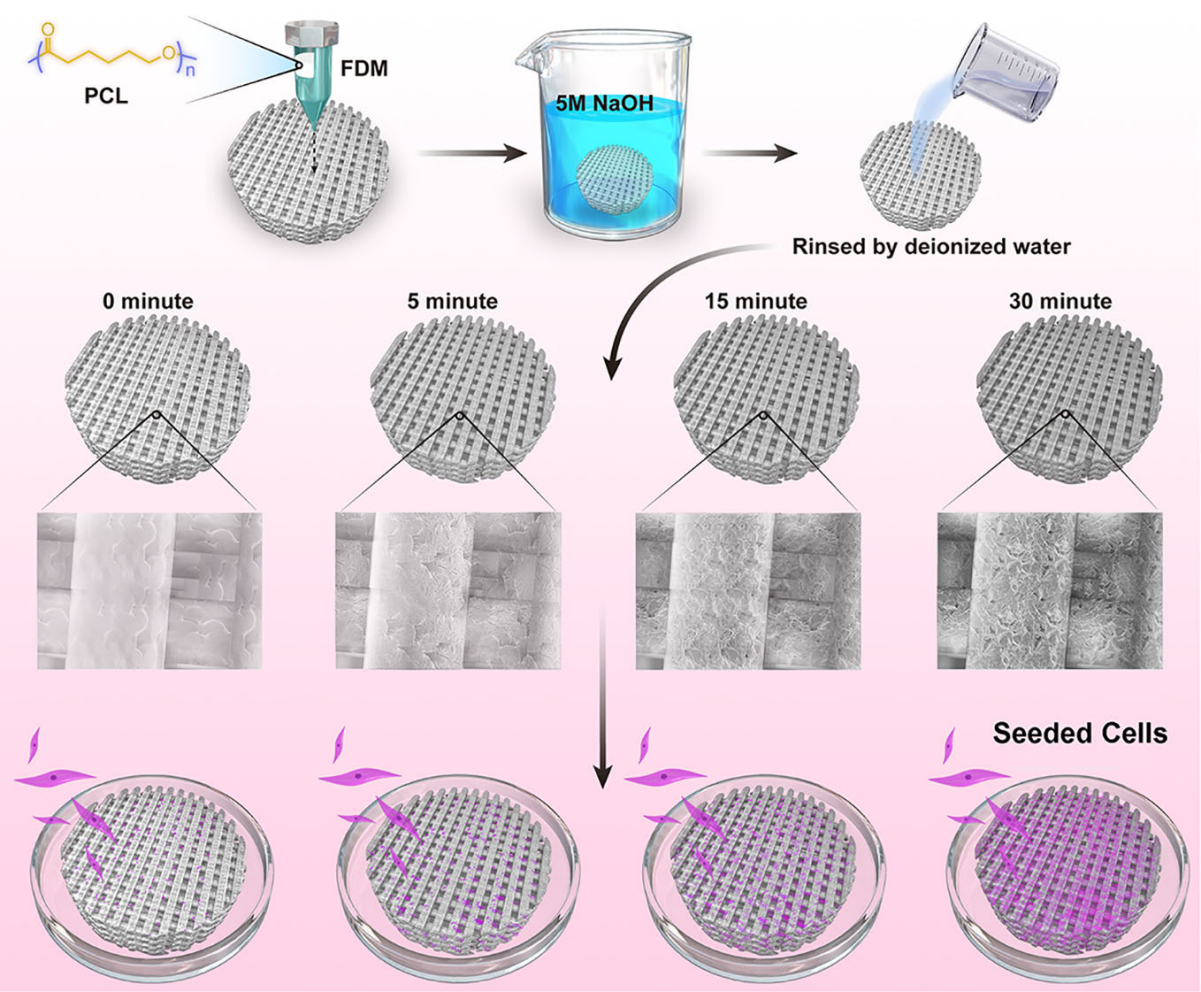
Schematic illustration for NaOH-modified poly(ε-caprolactone) (PCL) scaffolds seeded with mesenchymal stromal cells (MSCs) and meniscal fibrocartilage cells (MFCs).

In this work, we devised a simple and efficient strategy of NaOH etching to facilitate seeded cells to attach and proliferate on the PCL scaffolds (**Figure 1**). Based on the fused deposition modeling (FDM) technique, 3D PCL scaffolds were well fabricated with homogeneous microstructures, such as pore size, pore interconnectivity, and satisfactory mechanical properties. Adjusting the alkali soaking time of PCL scaffolds could significantly improve their hydrophilicity and increase the roughness of surfaces without severely impairing the biomechanical properties. Notably, the rougher surfaces of PCL fibers were beneficial to the attachment, viability, and proliferation of two commonly used seeded mesenchymal stromal cells (MSCs) and meniscal fibrocartilage cells (MFCs). Overall, this universal alkali soaking strategy could be a cost-efficient method of hydrophilic modification for the construction of cell-scaffold for meniscal regeneration.

## Materials and Methods

### Scaffold Fabrication and Hydrophilic Modification

3D-PCL (Mn=80,000, Sigma-Aldrich, MO, USA) scaffolds were fabricated by FDM 3D printing system as previously described (Zhang et al., 2017). Briefly, the PCL particles were added into a metal tube and melted to molten state before extrusion by a nozzle. During the fabrication process of layer-by-layer, the metal nozzle could lay down the PCL fibers at the 0°–90° direction controlled by computer-aided system. The printing parameters were set to ensure the microstructures of scaffolds to be dispersed homogenously, including pore size, thickness and space between two layers. After fabrication, the scaffolds were cut into some small cylinder examples by trephine. Those cylinder scaffolds were treated by 5 M NaOH for different time point at 0, 5, 15, and 30 min at room temperature. After soaking, the scaffolds were washed thoroughly with flowing water 12 h and rinsed by deionized water for three times.

### Characterization of 3D-Printed PCL Scaffolds With or Without Hydrolysis Treatment

After being coated with a 5-nm layer of gold in a high-vacuum gold sputter coater, the scaffolds were observed under scanning electron microscopy (SEM) (JEOL USA, Inc., Peabody, MA, USA) to observe the morphology of produced scaffolds treated and untreated with 5 M NaOH. In order to measure the microstructural parameters of scaffolds, we used Image-J software (NIH Image, Bethesda, MD) to analyze mean pore sizes and road widths of samples (*n* = 4). The Leica DCM 3D dual system (Leica, Nussloch, Germany) was used to display the surface topography and calculate the surface area roughness (Sa) of 3D PCL scaffold. Images were taken with a confocal objective with a magnification of 150×, and 10 independent sites of a sample under the same conditions were observed and analyzed (*n* = 3). The chemical characteristics of samples were revealed by ^1^H nuclear magnetic resonance spectrum (^1^H NMR) on 600 MHz Bruker AVII-600 spectrometers.

### Water Contact Angle Measurements

The water contact angle was evaluated for the change of hydrophilicity of untreated and treated PCL. Considering the existing porous structure of PCL scaffold, water would penetrate the pores leading to the failed results. Therefore, static contact angle measurements were performed on the produced PCL-based membranes prepared by 5wt% of PCL chloroform solution into a poly(tetrafluoroethylene) (PTFE) mold, which were then treated with NaOH, rinsed by deionized water with abovementioned method and dried in air for 24 h. Untreated PCL membranes were prepared for 0 min group. Contact angles of deionized water drops were recorded and calculated at room temperature with Drop shape Analyzer-DSA100 (Krüss, Hamburg, Germany) (Shen et al., 2008). Twelve independent determinations at different sites of three films' (Four sites were collected in each film, *n* = 3) surfaces were averaged.

### Degradation Experiments *In Vitro*

In this experiments, PCL scaffolds of all groups were completely immersed into phosphate buffered saline (PBS, pH=7.4) to investigate whether hydrolyzed effects applied by NaOH could accelerate the degradation rate of PCL. In brief, the dry weights of samples (*n* = 6) among all groups were recorded as *m_0_* before hydrolysis. After treatment by NaOH for different time, these samples were rinsed by distilled water thoroughly and immersed into PBS at room temperature whose dry weights were then recorded respectively as *m_1_* at the first day and fifteenth day after degradation in PBS. The mass loss (%) during immersion into PBS was calculated as the following formula.

$$\text{Mass loss \%}=\;\left[\frac{m_{0}-m_{1}}{m_{0}}\right]\times 100\%$$

### Mechanical Tests

To investigate the hydrolyzed effects mediated by 5 M NaOH on scaffolds' mechanical properties, we performed compression and tensile tests on the cylindrical samples and “dog-bone” shape samples of PCL, which were printed according to the same protocols and parameters. For compressive test, the cylindrical sample of 6 mm diameter and 2.5 mm thickness was prepared by trephine. The compressive test was conducted at a constant stress rate of 1 mm/min (*n* = 5). For tensile test, the “dog-bone” shape sample of 75 mm length and 2.5 mm thickness was loaded by tensile force to ultimate failure at a rate of 2 mm/min (*n* = 5).

### Biological Evaluation

#### Isolation, Culture of seeded cells

All protocols of animal experiments for isolation of MSCs and MFCs were approved by the Animal Care and Use Committee of Peking University Third Hospital. The method we used to isolate, culture and passage seeded cells was similar to the previous article published by our group (Wang S. et al., 2016). MSCs and MFCs reaching 80% to 90% confluence were trypsinized with 0.25% trypsin/0.1% ethylene diamine tetraacetic acid (EDTA, Gibco BRL Co. Ltd. Gaithersburg, MD, USA) for passage at 1:2. MFCs and MSCs were used between the second passage (P2) and the fifth passage (P5).

#### Cell Seeding

Scaffolds were totally immersed into 70% ethanol for 12 h to sterilize before they were rinsed in PBS three times, and then air-dried for 24 h at room temperature in a biosafety cabinet. The scaffolds were wet with 200 µl of media containing 10% fetal bovine serum (FBS) and kept in standard conditions (37°C under 5% CO_2_ and 95% humidity) for 2 h prior to cell seeding. Cells were seeded on each scaffold (50 µl of medium containing around 5 × 10^4^ cells). The cell-seeded scaffolds were incubated at standard conditions (37°C under 5% CO_2_ and 95% humidity) for 2 h to allow cell attachment before the addition of 1 ml of culture media.

#### Cell Viability/Proliferation

We adopted the alamarBlue™ Cell Viability Reagent (Life Technologies, Carlsbad, CA, USA) to study the cell proliferation on the scaffold untreated and treated with NaOH. Briefly, cell proliferation was measured at the first and fifth day after cell seeding to PCL scaffolds, the cell-seeded PCL scaffolds were transferred to a new 48-well plate, and 10% alamarBlue™ solution (alamarBlue™: growth culture medium, 1:9; v/v) was added to each well and the control well. The plates were incubated for 4 h under standard conditions. After incubation, 150 µl of each sample was pipetted and moved to a 96-well plate, and the absorbance was measured at 570 and 600 nm respectively with a plate reader (*n* = 5). The cellular viability was assessed with a LIVE/DEAD Viability/Cytotoxicity assay (Invitrogen, Carlsbad, CA, USA) under Leica TCS-SP8 confocal laser microscopy (CFLM; Leica, Nussloch, Germany). All PCL scaffolds with seeded cells were cultured in complete medium for 3 days. Before the test, the samples (*n* = 3) were washed in PBS three times, followed by immersion in 500.0 µl of PBS with 2.0 mM calcein AM and 4.0 mM ethidium homodimer-1 reagents, and incubated for 30 min at room temperature. Excitation wavelength of 568 or 488 nm was adopted to excite the fluorescence.

#### Cell Morphology and Attachment

The cell morphology and attachment were observed under SEM. After a three-day culture, cell-seeded scaffolds (*n* = 3) were rinsed by PBS and fixed immediately in a 2.5% glutaraldehyde solution, and then dehydrated with a gradient ethanol and the critical point drying was performed in liquid CO_2_ at 37°C. After being coated with gold, those samples were viewed by a scanning electron microscope. F-actin and nuclear of the cells in the 3D scaffolds (*n* = 3) cultured in 3 days after seeding were observed by confocal microscopy. Brieﬂy, the samples were washed with PBS three times and fixed with 4% paraformaldehyde for 30 min and the cellular membranes were penetrated with 0.5 % Triton X-100. The cytoskeleton was stained by Acti-stain^TM^ 488 phalloidin (100 nM; Cytoskeleton Inc., Denver, CO, USA) for 30 min at room temperature, and the nuclei were stained by RedDot^TM^-2 (Cambridge Bioscience; #40061) working solution for 10 min.

### Statistical Analysis

The data in the current work were expressed as means ± standard deviation (SD). We used SPSS statistical software (Version 20.0; SPSS Inc., Chicago, IL, USA) to perform datum analysis. A one-way analysis of variance (ANOVA) and the paired sample *t* test were used to analyze the results, *p* < 0.05 was considered statistically significant.

## Results and Discussion

### Characterization of 3D PCL Scaffold

**Figure 2:**
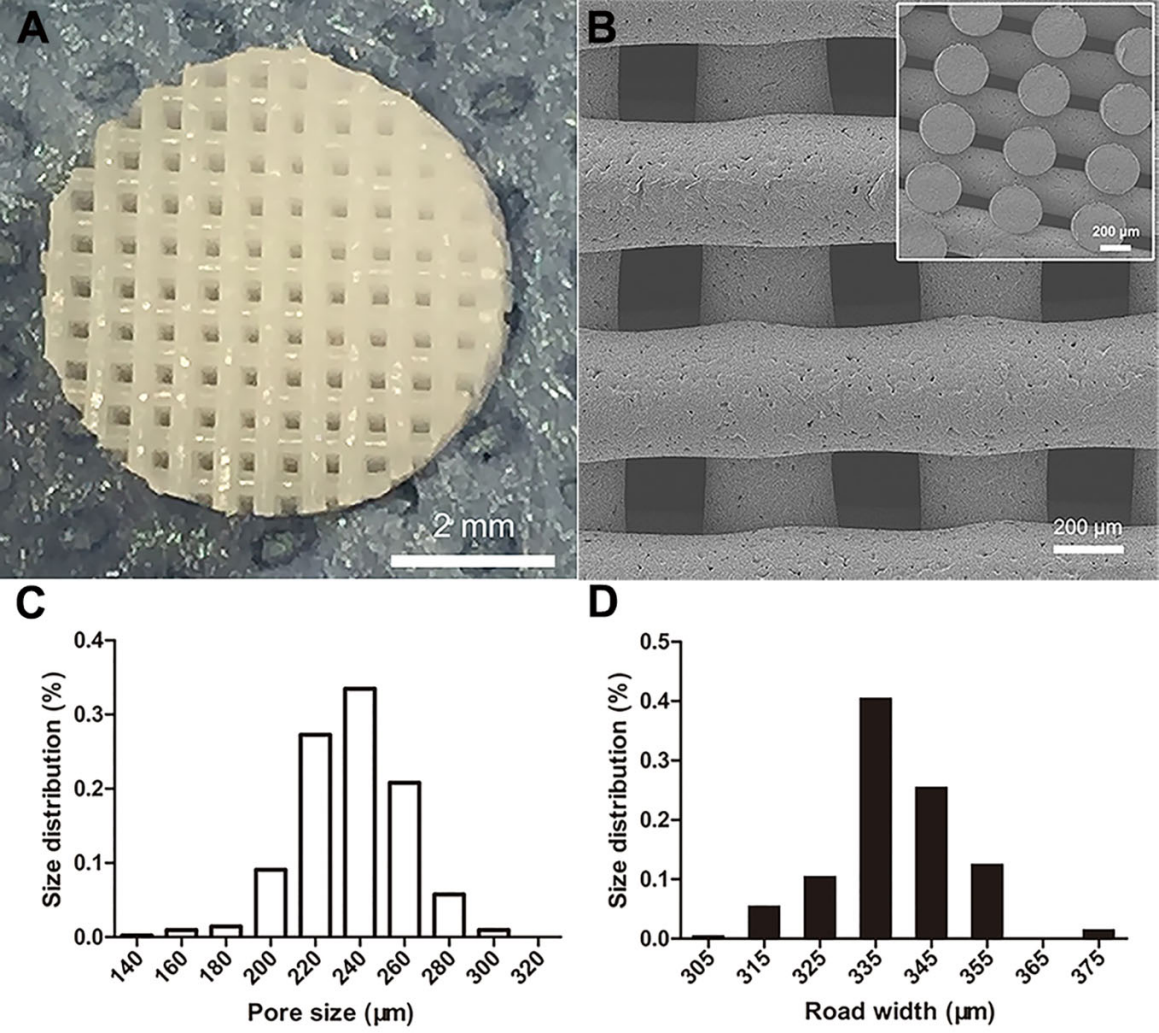
Morphological characteristics of poly(ε-caprolactone) (PCL) scaffolds. The PCL specimen with 6.0 mm in diameter and 2.5 mm height, scale bar represented 2 mm **(A)**. The SEM image of top surface and cross-section of scaffold, scale bar represented 200 µm **(B)**. The distribution of PCL scaffolds' micropore size **(C)** and road width **(D)**.

**Figure 3:**
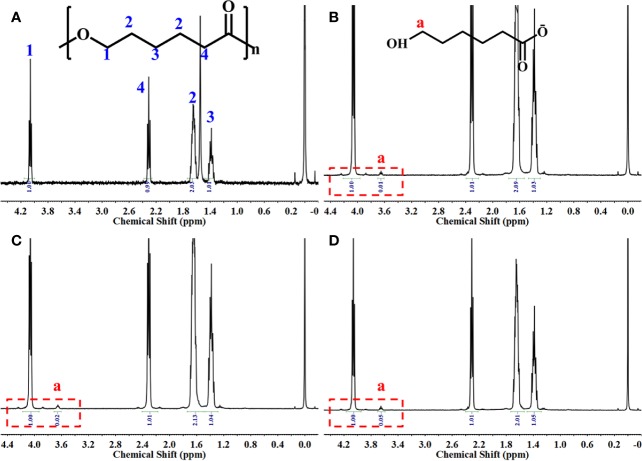
^1^H-NMR spectrum of the samples. Original poly(ε-caprolactone) (PCL) scaffolds **(A)**, PCL scaffolds etched by NaOH for 5 **(B)**, 15 **(C)**, and 30 min **(D)**.

The 3D PCL scaffold was printed by FDM technique with 30 mm×30 mm×2.5 mm, and then trimmed into cylindrical test samples with a 6-mm diameter corneal trephine respectively for the following experiments. The gross view of cylindrical test sample with 6.0 mm in diameter and 2.5 mm height was illustrated in **Figure 2A**. The PCL fibers deposited layer by layer along 0°–90°, forming the micropores with square geometry, in which the top surface and cross-section of PCL fibers displayed the relatively homogenous microstructures and interconnection by the SEM observation (**Figure 2B**). We also analyzed the distribution of pore size and road width and found that the mean pore sizes of scaffolds were 236.5 µm ± 23.8 µm with the arrangement from 146.7–300.0 µm while the mean road widths were 338.1 µm ± 11.6 µm with the arrangement from 306.7–370.9 µm (**Figures 2C, D**). FDM, a 3D printing technique, melts, extrudes biomaterials and then deposits the examples with targeted geometric shape by controlling the pathway of moving nuzzle. In our previous work, we investigated the role of mean pore sizes of PCL-fabricated meniscal implants printed by FDM in promoting the meniscal regeneration (Zhang et al., 2016). As a cost-effective rapid prototype technique, it not only avoided using the toxic organic solvents for materials during the process of fabrication, but also generated relatively homogenous microstructural parameters, such as pore sizes, porosities, and road widths of fibers, reducing the differences among testing specimens effectively (Do et al., 2015). The change in chemical structures of PCL scaffolds immersed into NaOH was detected by NMR as illustrated in **Figure 3**. Compared with the original PCL, an obvious signal peak occurred at 3.7 ppm in the ^1^H-NMR spectra, which belongs to the free hydroxyl group at the end of PCL chain, indicating the breakage of ester bond and hydrolysis of PCL under alkali conditions. In addition, the quantitative proof was clearly seen in the integral areas ratio (1:a). Along with the immersion time in NaOH for 5, 15, and 30 min, this typical area ratio was 100:1, 50:1, and 20:1, respectively, which further reflected more hydrophilic function groups exposing at the surfaces of samples as they soaked into NaOH for a longer time. Similarly, all of other samples showed the same characteristics, revealing that the hydrolyzed effect did not change the bulk chain of PCL and alkali treatment was one of the suitable strategies for PCL surface hydrophilic modification.

### Water Contact Angle and Morphological Observation of PCL

**Figure 4:**
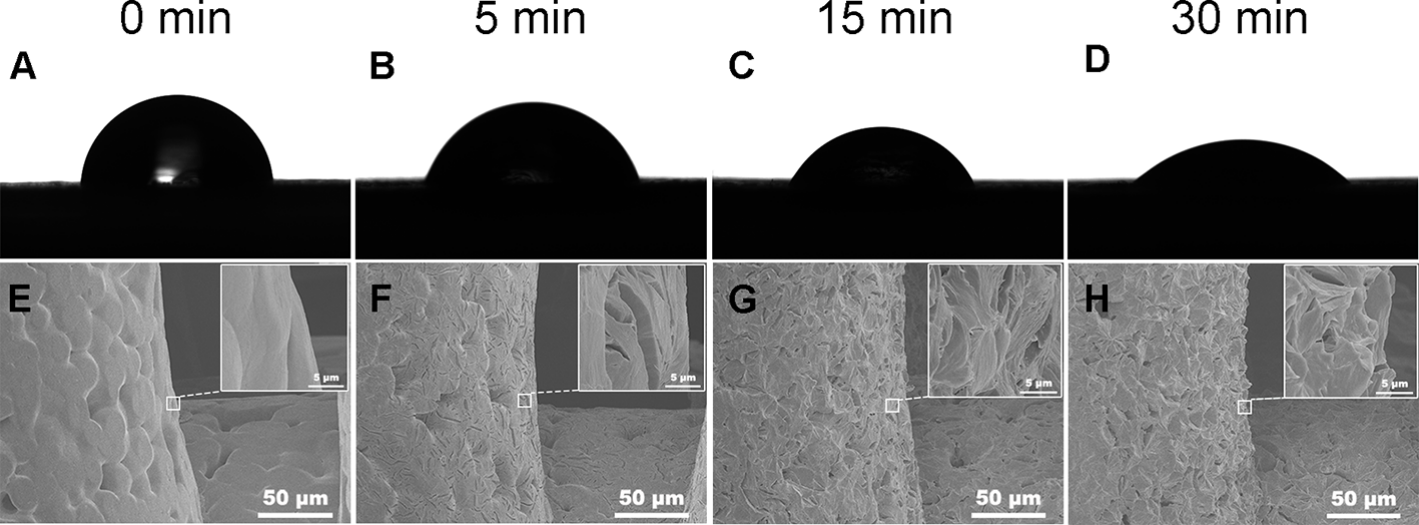
Water contact angle in the air of poly(ε-caprolactone) (PCL) film. Samples treated by 5 M NaOH for different time **(A–D)**. Scanning electron microscopy (SEM) images of PCL scaffolds' surfaces soaked in 5 M NaOH for different time **(E–H)**. **(A, E)** 0, **(B, F)** 5, **(C, G)** 15, and **(D, H)** 30 min.

**Figure 5:**
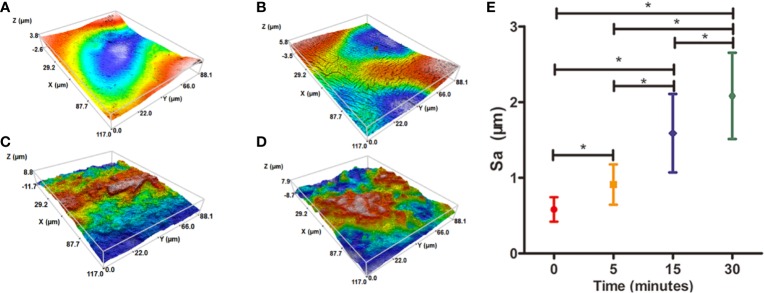
Three-dimensional (3D) confocal topographical images of poly(ε-caprolactone) (PCL) scaffolds' surfaces treated by 5 M NaOH at different time point. **(A)** 0, **(B)** 5, **(C)** 15, and **(D)** 30 min. The results of average roughness values (Sa) of PCL scaffolds' surfaces treated by 5 M NaOH at different time point, *P < 0.05 **(E)**.

Water contact angel in air could illustrate the hydrophilicity of material surface, thus, it is a direct indicator of the hydrophilic change of PCL surface before and after treatment of NaOH solutions. However, because of the porous texture of PCL scaffold, it is hard to measure the values directly. Hereby, we characterized the hydrophilic changes of films paved by the same materials and exposed into the 5 M NaOH under the same condition with scaffold to assess the hydrophilic modification result of PCL after NaOH treatment. The results of apparent static contact angles on PCL films soaked in 5 M NaOH at different time point were illustrated in **Figures 4A–D**. After the treatment of NaOH, the water contact angles of PCL films in 5, 15, and 30 min of treatment groups was 66° ± 4.4°, 55° ± 4.2°, and 39° ± 5.7°, respectively, displaying a significant reductive tendency compared to the untreated PCL films with 83° ± 7.1°. A statistical difference was detected among all alkali-treated PCL films at different time point, suggesting that the hydrophilicity of PCL surface increased with the soaking time prolonging in 5 M NaOH. (*p*<0.05). Due to the hydrolysis of aliphatic polyester in NaOH solutions, hydrophilic carboxyl and hydroxyl of PCL surface can be produced by cleaving the ester bonds (Wang W. et al., 2016). When the PCL-based films were soaked into 5 M NaOH for different time, the water contact angle variation indicated that the alkali treatment could effectively degrade the ester bond and dramatically etch the PCL surface to improve its hydrophilicity that was in line with the previous research (Yang et al., 2003). These results also suggested that 30 min soaking in 5 M NaOH is enough for PCL (Mn = 80000) to improve its hydrophilicity without prolonged soaking time. The SEM images in **Figures 4E–H** revealed the change of surfaces' characteristics of PCL scaffolds. 3D confocal topographical images in **Figures 5A–D** represented and analyzed the roughness of 3D scaffolds' surfaces treated by NaOH for 0, 5, 15, and 30 min. Evidently, the roughness increased dramatically because of the different treatment time soaked in NaOH solution as showed in **Figure 5E** (*P*<0.05). The surface of untreated PCL scaffold was relatively smooth, however, the surfaces crazed gradually with the increase of gaps, cracks, and defects, which even appeared volcanic topographical characteristics onto scaffolds etched by NaOH for 30 min. These results demonstrated that controlling the etching time of NaOH could not only modify the hydrophilicity of PCL, but also regulate the surfaces' roughness.

### Degradation Experiments *In Vitro*

**Figure 6:**
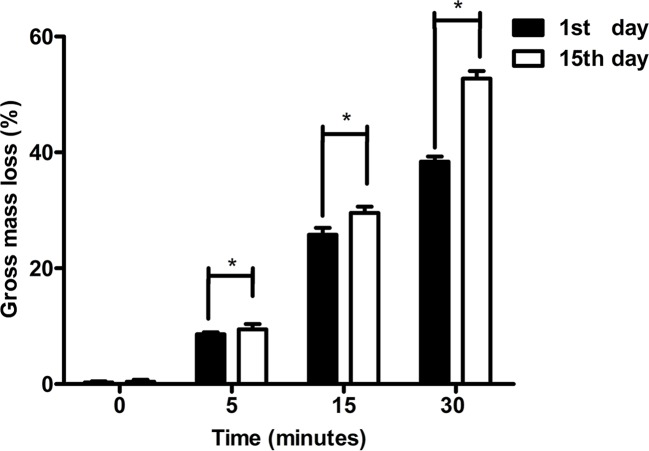
Degradative experiments of poly(ε-caprolactone) (PCL) scaffolds. The change in mass loss (%) of PCL scaffolds modified by 5 M NaOH at different time point was detected at 1^st^ day and 15^th^ day. The results were analyzed by the paired sample *t* test, *P < 0.05.

The results of degradative changes in mass loss (%) of original and hydrolyzed PCL scaffolds were shown in **Figure 6**. At the first day, the mass loss % of PCL scaffolds etched by NaOH for 0, 5, 15, and 30 min was 0.27%±0.19%, 8.58%±0.37%, 25.79%±1.2%, and 38.38%±0.91%, respectively. After immersion into PBS for fifteen days, the mass loss % of those samples was 0.40%±0.35%, 9.43%±0.95%, 29.57%±1.08%, and 52.77%±1.30%, respectively. The mass loss of all samples among groups of 5, 15, and 30 min at the fifteenth day showed higher than their loss at the first day (*p* < 0.05), while no significant results were detected among untreated scaffolds between the first and second measurements (*p* > 0.05). Apparently, PCL was much more easily degraded *in vitro* after exposure to 5 M NaOH, which indicated that the surface modification of alkali applied on PCL could not only improve its hydrophilicity, but also accelerate its degradation. The main mechanism related to abovementioned phenomenon is the formation of hydroxyl and carboxyl groups at the surfaces of scaffolds. When the increasing number of hydrophilic groups exposed with materials soaked into alkali solutions much longer, more hydrogen bonds between the surfaces of materials and water molecules tied and contributed to rapid degradation of PCL, forming a positive feedback during degradation process (Gupta et al., 2019).

### Mechanical Properties of 3D-Printed PCL Scaffolds

**Figure 7:**
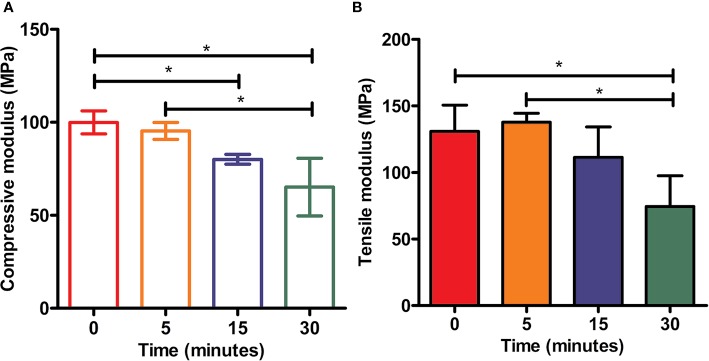
Mechanical tests of scaffolds. Static compressive moduli **(A)** and tensile moduli **(B)** of three-dimensional (3D) poly(ε-caprolactone) (PCL) samples under the treatment of NaOH for different time, *P<0.05.

The mechanical properties (Yong's moduli) of tested samples among all groups were presented in **Figure 7**. The compressive modulus of each example was calculated as the ratio of stress to strain, i.e., the slope of the stress–strain curve. As shown in **Figure 7**, PCL without etching by NaOH possessed strongest mechanical properties compared to other scaffolds in the rest groups with treatment of 5 M NaOH for 5, 15, and 30 min respectively, but no statistical difference was observed from the scaffolds in 5 min group (*p* > 0.05). PCL scaffolds soaked in 5 M NaOH for 30 min displayed the weakest compressive moduli compared to other scaffolds exposed to alkali solution, but no differences of compressive moduli were detected from scaffolds in NaOH for 15 min (*p* > 0.05). The results of tensile tests were similar to the compressive ones. Compared with PCL samples in 0 and 5 min group, the tensile moduli of scaffolds hydrolyzed by NaOH for 30 min showed the lowest ones (*p* < 0.05). Because of the hydrolyzed effect of alkali on PCL, solid content of PCL scaffold would be decreasing and bulk polyester would be degrading during the period of contacting with NaOH solution, which would inevitably compromise scaffolds' mechanical properties and alter the mechanical integrities. Fortunately, compared with the compressive moduli of the native meniscal tissues, the compressive moduli of PCL scaffolds treated by NaOH for 30 min were still higher, such as the maximum instantaneous compressive modulus of native human's meniscus was around two MPa reported in the previous research (Fischenich et al., 2015). It is, therefore, still acceptable about NaOH treatment within 30 min to satisfy the usage for PCL scaffolds.

### Cellular Proliferation

**Figure 8:**
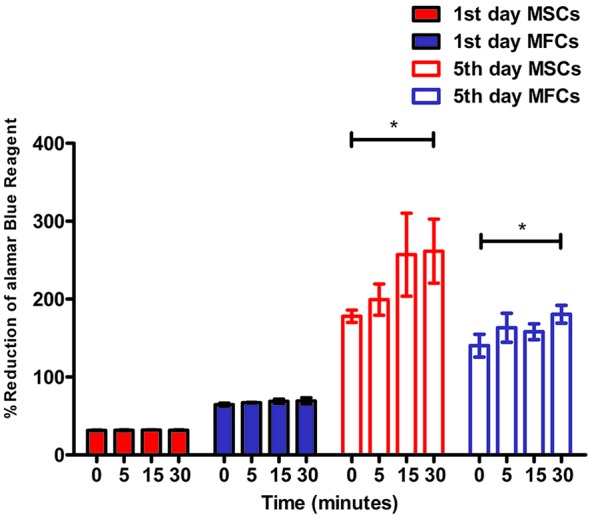
Cellular proliferation of mesenchymal stromal cells (MSCs) and meniscal fibrocartilage cells (MFCs) onto scaffolds. Scaffolds untreated or treated by NaOH for different time (0, 5, 15, and 30 min) at 1^st^ day and 5^th^ day after cell seeding, *P < 0.05.

The alamarBlue™ assay demonstrated that all constructs with cells (MSCs and MFCs) showed an increasing proliferative tendency during 5 days of *in vitro* culture (**Figure 8**). However, the percent of reduced alamarBlue™ of cell-scaffolds constructs among all groups on day 1 did not display significant increase (*p* > 0.05). On day 5, the percent of reduced alamarBlue™ of scaffolds treated by 5 M NaOH for 30 min with MSCs and MFCs was higher than those untreated constructs (*p*<0.05). Besides, both MSCs and MFCs in the other treated scaffolds did not show any proliferative advantage than untreated ones. Proliferative assay proved that the scaffolds with the longest soaking time in NaOH provided the best proliferative effect on MSCs and MFCs. This might be attributed to hydrolyzed effect of NaOH on PCL scaffolds, because hydrophilic function groups such as carboxyl and hydroxyl groups were exposed on the surfaces of scaffolds after treatment by NaOH. These generated functional groups might offer the adhesive recognition for seeded cells that could attach onto scaffolds rapidly and further proliferate onto them. Besides, the rougher surfaces could account for another reason why both MSCs and MFCs showed better proliferation in the scaffolds treated by NaOH for 30 min. The increasing roughness of materials' surfaces meant the larger surfaces' areas, which could extensively support more seeded cells surviving onto the PCL scaffolds.

### Cellular Viability, Morphology, and Attachment

**Figure 9:**
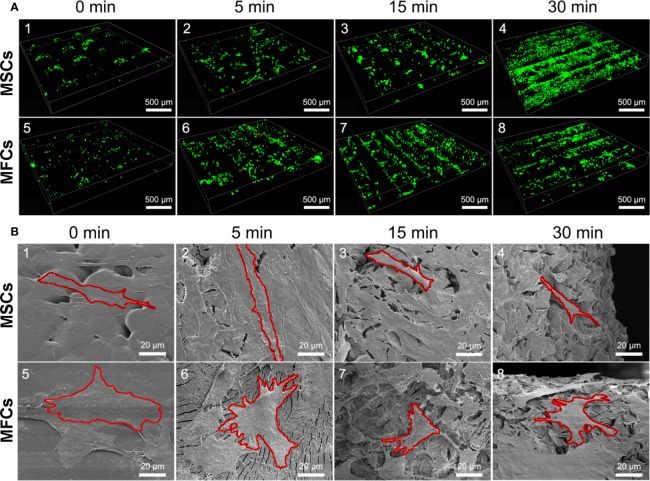
Images of seeded cells on scaffolds detected by laser confocal microscopy and scanning electron microscopy (SEM). **(A)** Confocal images of LIVE/DEAD assay presented the cellular viability and distribution onto the PCL scaffolds at 3^rd^ day (green, live cells, red, dead cells.). **(B)** SEM images of cellular attachment and morphology onto the surfaces of PCL scaffolds at 3^rd^ day, red outline was added and used to isolate and label single cell in these pictures.

**Figure 10:**
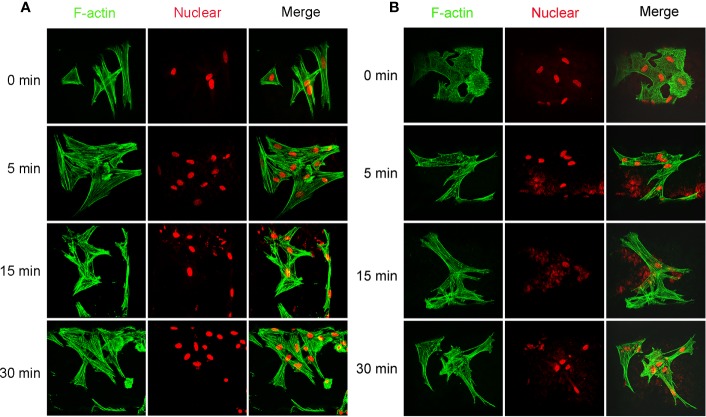
Images of cellular skeleton and nuclear. Images of F-actin stained with Alex 488 and nuclear stained with Rot-2 of mesenchymal stromal cells (MSCs) **(A)** and meniscal fibrocartilage cells (MFCs) **(B)** seeded on the poly(ε-caprolactone) (PCL) scaffolds modified with or without 5 M NaOH, which were taken by confocal laser microscopy.

Cellular viability of seeded cells in the scaffolds untreated or treated by NaOH was evaluated *via* laser confocal microscopy after 48 h of culture. LIVE/DEAD stain showed the live cells (green) or dead cells (red) in the scaffolds. As shown in **Figure 9A**, live MSCs and MFCs were spreading and distributing on the surfaces of PCL scaffolds along the aligned PCL fibers. 3D rendering of cell-PCL constructs displayed that the number of live MSCs and MFCs on the scaffolds treated by NaOH were larger than those were on the untreated ones in a qualitative way. Especially, both MSCs and MFCs have been forming the confluent cell sheet on PCL scaffold soaked in NaOH for 30 min to portrait the structure and pore shape of scaffold clearly. Scaffolds seeded with MSCs and MFCs were observed by SEM to evaluate the cellular attachment and morphology. Extensive and firm cellular attachment was observed as shown in **Figure 9B**. Both MSCs and MFCs were spreading and distributing on the surfaces of PCL fibers, forming the interconnections among the cells. These images also reflected that MSCs appeared vary shapes, like elongated, polygonal, or spindle morphologies, which were similar to the MFCs. Successful cellular attachment suggested that the modified-PCL scaffolds could not bring about the negative effect on the survival and proliferation of cells. Generally, it was believed that hydrophilic surfaces favor cell adhesion and growth (Chen et al., 2014), which was confirmed by LIVE-DEAD assay after 48-h culture *in vitro*. Both MSCs and MFCs showed better distribution and viability in the scaffolds after treatment with NaOH solution for 30 min, compared with the other groups. Particularly, more live cells were observed on the scaffolds soaked in NaOH for 30 min. Although cell's, spreading and attachment were observed by SEM among all groups, MSCs and MFCs distributing on the surfaces of scaffolds treated by NaOH were bridging with each other and more aggregative on the scaffold filament. Besides, the area of attached single cell on the untreated scaffold was larger, leading to the loss of typical morphology of both MSCs and MFCs such as prolonged, polygons, and spindle-liked shape. The morphology of F-actin stained with Alex 488 also displayed that the slightly different shape of MSCs and MFCs seeded on the scaffolds among all groups, as shown in **Figure 10**. These phenomena indicated that transformed surface's morphology of scaffold produced by NaOH treatment had an impact on the cells surviving on its surface, which might then affect the phenotype of seeded cells and their functions. It has been reported by some studies that surface roughness of biomaterials, except for hydrophilicity, could favor the cell adhesion (Chen et al., 2014; Faia-Torres et al., 2014; Zhou et al., 2015). In other words, increased interface roughness could account for the differences of cellular proliferation and viability among scaffolds treated or untreated by NaOH.

## Conclusions

In summary, we established a simple and universal method on the improvement of hydrophilicity of PCL scaffold. By means of the FDM technique and the subsequent NaOH treatment method, 3D PCL scaffolds were fabricated with uniform pore size and homogeneous pore interconnectivity and modified for wettability. This simple alkali-soaking modification not only improved the hydrophilicity, but also produced the rougher surface of PCL fiber that might maintain the typical cellular morphology, which furnished the two commonly used MSCs and MFCs with better cell attachment, viability, and proliferative capacities on the scaffold. Thus, we believe that the extrusion-based additive manufacturing system could be a promising platform for the fabrication of novel and smart scaffold, and alkali-soaking treatment could be a cost-efficient strategy on hydrophilic modification for construction of cell-scaffold for meniscal regeneration.

## Data Availability Statement

The datasets generated for this study are available on request to the corresponding authors.

## Ethics Statement

The animal study was reviewed and approved by Animal Care and Use Committee of Peking University Third Hospital.

## Author Contributions

Z-XZ and Y-RC contributed to this study: conception and design, collection and assembly of data, analysis and interpretation of data, drafting of the manuscript. DJ and FY: assistance in analysis and interpretation of data. W-BJ, J-YZ, F-ZY, and Z-MM: technical support on analysis and interpretation. J-KY and XW: conception and design, critical revision, and final approval of the manuscript.

## Funding

This study was supported by the National Natural Scientific Foundation of China (Grant No. 31670982, Grant No. 81630056, Grant No. 51773004 and Grant No. 51973226), the Open Research Project Funds (K2019-27), and the National Key Research and Development Program of China (Grant NO. 2016YFC1100704).

## Conflict of Interest

The authors declare that the research was conducted in the absence of any commercial or financial relationships that could be construed as a potential conflict of interest.
